# Electrocardiographic markers predict hemodynamic parameters in adults with uncorrected secundum atrial septal defect

**DOI:** 10.1186/s43044-024-00596-x

**Published:** 2025-01-10

**Authors:** Kunti N. Umamy, Astri K. Martiana, Risalina Myrtha, Irnizarifka Irnizarifka, Alfa A. Nursidiq

**Affiliations:** Department of Cardiology and Vascular Medicine, Faculty of Medicine, Sebelas Maret University, Surakarta, Indonesia

**Keywords:** Secundum ASD, Electrocardiography, RAP, PVR

## Abstract

**Background:**

Precapillary pulmonary hypertension (PH) as complication in atrial septal defect (ASD) is closely related to right heart hemodynamics, such as right atrial pressure (RAP) and pulmonary vascular resistance (PVR). Right heart catheterization (RHC) as the gold standard for their measurement is invasive and not widely available in Indonesia. Electrocardiography (ECG) was proposed to be alternative in this matter.

**Method:**

This is a retrospective observational study with cross-sectional design. We collected data and measured ECG parameters of secundum ASD patients who underwent elective RHC from May 2019 until November 2023. We compared several ECG parameters based on RAP (< 8 and ≥ 8 mmHg) and PVR (< 5 and ≥ 5 WU).

**Result:**

Eighty-three patients were included. The R_V1_ was the only ECG marker that showed significant difference based on RAP (AUC 0.639, sensitivity 61.7%, specificity 61.1%, p = 0.030) and PVR (AUC 0.801, sensitivity 73.2%, specificity 81%, p < 0.001). Several ECG parameters were found significantly different based on PVR value only, namely S_V5_ (AUC 0.773, sensitivity 80.5%, specificity 71.4%, p < 0.001), S_V6_ (AUC 0.823, sensitivity 80.5%, specificity 81%, p < 0.001), right ventricular Sokolow–Lyon index (RVSLI) (AUC 0.841, sensitivity 82.9%, specificity 83.3%, p < 0.001), R/S_V1_ (sensitivity 97.6%, specificity 16.7%, p = 0.031) as well as right ventricular strain (sensitivity 87.8%, specificity 69%, p < 0.001). Multivariate regression analysis showed RVSLI (OR 15.66 (4.46–55.02), CI 95%) and right ventricular strain pattern (OR 9.23 (2.43–35.14), CI 95%) had the best predictive value for PVR ≥ 5 WU.

**Conclusion:**

In adults with secundum ASD, several ECG markers have potential role in predicting PVR ≥ 5 WU with satisfying sensitivity and specificity, but not in predicting RAP.

**Supplementary Information:**

The online version contains supplementary material available at 10.1186/s43044-024-00596-x.

## Background

Based on recent study, it is estimated that around 40,000 babies are born with congenital heart disease (CHD) in Indonesia. Approximately 1.5 million adults in Indonesia currently live with CHD [1]. Secundum ASD is the most common type of ASD worldwide. Although it is known as relatively simple defect, there are still many challenges in optimizing its diagnosis and therapy [2]. Uncorrected ASD may increase patient morbidity and mortality risk, along with patients’ age and mainly related to right heart failure and PH [3]. Mostly, ASD was diagnosed and corrected during childhood in developed countries (like in Europe or the USA). However, in developing country such as Indonesia, ASD were mostly remained undiagnosed until adulthood where those patients commonly have developed PH.

Precapillary PH in ASD is closely related to right heart hemodynamics. Currently, several stratification systems have been developed in order to assess mortality risk and disease severity in patients with pulmonary arterial hypertension (PAH). Cardiopulmonary hemodynamic examination provides prognostic information that has been proven to be accurate, both at first diagnosis and at follow-up. Survival risk stratifications in PAH such as REVEAL score and ESC/ERS stratification system also involve hemodynamic variables, namely RAP and PVR [4, 5].

Right heart catheterization is the gold standard for RAP and PVR measurements, which is difficult to be performed routinely. Apart from being invasive, the facility needed to perform RHC is not widely available in Indonesia. In contrast, ECG examinations are widely available, safe and easy to perform. Several studies have been carried out correlating RA and RV parameters from ECG with hemodynamic parameters in patients with PH or CHD [6–9]; however, their results were conflicting and none have linked ECG parameters with RAP and PVR specifically in adults with uncorrected secundum ASD so far.

## Method

This is a retrospective, observational study with cross-sectional design that was conducted in Dr. Moewardi general hospital. Data of all patients > 18 y.o with uncorrected secundum ASD who underwent elective RHC from May 2019 until November 2023 were collected. All standard 12-lead ECG was performed while patients in supine position by cardiovascular nurse < 24 h prior to RHC. Exclusion criteria included other concomitant congenital or valve defects beside tricuspid regurgitation, basic cardiac rhythm other than sinus, unstable hemodynamic, sign and symptoms of acute heart failure, moderate to severe pericardial effusion, left ventricle ejection fraction (LVEF) < 50% and history of chronic obstructive pulmonary disease (COPD).

Numeric data of ECG parameters were measured using ImageJ (NIH, USA) on 3 continuous beats in the same lead; then, the average was taken. Measurement and assessment were carried out by 2 cardiologists who were blinded to patients’ data. Analyzed ECG parameters included P wave morphology in V1 (mitral, pulmonal, normal), P wave amplitude in lead II (P_II_), QRS duration in V1 (QRS_V1_), R wave amplitude in V1 (R_V1_), R/S ratio in V1 (R/S_V1_), S wave amplitude in V5 and V6 (S_V5_, S_V6_), RVSLI (by adding R_V1_ and deepest S in V5 or V6), PR interval (PR_int_) and PR segment depression (PR_dep_) in lead II, RV strain pattern and QRS axis. All of these parameters were compared based on RAP (< 8 mmHg and ≥ 8 mmHg) and PVR (< 5 WU and ≥ 5 WU) from RHC.

The definition of P wave morphology in this study was based on previous study conducted by Mirtajaddini et al., P wave morphology in V1 defined as P mitral if the negative deflection  ≥ 1 mm with duration of ≥ 0.04 s, P pulmonal if positive deflection  ≥ 1 mm, and normal if the P wave amplitude deflection < 1 mm. PR_dep_ defined as PR segment depression ≥ 1 mm in lead II [10]. RV strain, defined as ST segment depression or T wave inversion in right precordial leads V1-V3 or V4, might be accompanied with RBBB pattern and right axis deviation.

### Statistical analysis

We performed interrater reliability of ECG measurement by using Cohen’s kappa for categorical and intraclass correlation coefficient (ICC) for continuous variable. Univariate analysis was performed using Chi-squared or Fisher exact tests for qualitative and Mann–Whitney or Independent t test for comparing continuous variables. We obtained ROC curves to determine cut-off value and AUC of ECG parameters. Bivariate analysis was performed using Chi-squared to obtain sensitivity, specificity and prevalence odds ratio (OR). Variables with p value < 0.2 entered multivariate analysis. All statistical analysis were carried out by using SPSS software version 24 (Chicago, IL, USA); p value < 0.05 was considered statistically significant.

## Result

### Baseline characteristic

A total of 98 adults with uncorrected secundum ASD underwent elective RHC between May 2019 and November 2023. Fifteen patients excluded from analysis (7 patients had AF, 5 patients had moderate to severe stenosis pulmonal, 1 patient had LVEF < 50%, and 2 ECGs performed in non-standardized calibration); therefore, 83 patients enrolled in the current study. Of all those patients, 89% patients were females with median age of 32 y.o (17–64) and 89.1% had developed PH. Median diameter of secundum ASD was 2.5 and 2.6 (1.1–4.2) cm in study groups. During this study period, there were only 27 (32%) patients who had ASD closure, either by device or surgery. The rest were not eligible due to refractory PH or rejected to undergo surgery. Table 1 presents baseline and clinical characteristic of study population based on RAP and PVR.

**Table 1:** Clinical characteristics of study population based on RAP and PVR

Variables	RAP < 8 mmHg (n = 36)	RAP ≥ 8 mmHg (n = 47)	p value	PVR < 5 mmHg (n = 42)	PVR ≥ 5 mmHg (n = 41)	p value
Female^a^	74 (89.2%)	33 (91.7%)	0.725	36 (85.7%)	38 (92.7%)	0.483
Age^b^	30.5 (18–61)	33 (17–64)	0.613	36 (17–61)	30 (21–64)	0.154
BMI^b^	21.25±2.30	21.25±2.17	0.988	21.57±1.96	20.92±2.43	0.182
Hemoglobin^c^	13.45 (11.1–16.7)	13,8 (10.4–19.3)	0.468	13.1 (10.4–16.2)	14.5 (11.7–19.3)	0.001*
Hematocrit^c^	40.5 (33–48)	41 (34–61)	0.359	40 (33–50)	43 (34–61)	<0.001*
Creatinine^c^	0.7 (0.5–1.3)	0.8 (0.5–1.2)	0.861	0.7 (0.5–1.1)	0.8 (0.5–1.3)	0.733
Echocardiographic Parameters						
Defect diameter^b^	2.6 (1.2–3.7)	2.5 (1.1–4.2)	0.755	2.6 (1.1–4.2)	2.6 (1.6–4.2)	0.169
RVIDd^c^	3.7 (2.1–5.7)	3.7 (2.2–6.1)	0.442	3.7 (2.0–6.1)	3.7 (2.1–5.1)	0.030*
TAPSE^b^	2.51±0.46	2.27±0.44	0.019*	2.48±0.48	2.26±0.41	<0.001*
TR velocity maximum^b^	2.35±1.05	3.65±1.11	0.21	2.96±0.93	4.10±0.94	0.009*
LVEF^c^	66 (54–85)	65 (57–87)	0.916	63.5 (54–85)	68 (57–87)	
PH Probability^a^			0.417			<0.001*
Low	8 (22.2%)	8 (17.0%)		12 (28.6%)	4 (9.8%)	
Moderate	11 (30.6%)	10 (21.3%)		18 (42.9%)	3 (7.3%)	
High	17 (47.2%)	29 (61.7%)		12 (28.6%)	34 (82.9%)	
RLHC Parameters						
RAP^c^	6 (3–7)	11 (8–24)	<0.001*	8 (4–24)	9 (3–18)	0.288
PVR^c^	3.7 (0.19–25.37)	6.59 (0.08–45.86)	0.468	1.34 (0.08–4.54)	13.81 (6.17–45.86)	<0.001*
PVRI^c^	4.05 (0.12–17.03)	4.55 (0.27–48.48)	0.689	0.94 (0.12–5.79)	9.76 (4.24–48.48)	<0.001*
RVSP^c^	62.5 (22–139)	87 (32–143)	0.045*	47.5 (22–121)	104 (32–143)	<0.001*
PCWP^c^	7 (3–19)	11 (7–20)	<0.001*	9 (4–18)	9 (3–20)	0.385
mPAP^c^	34.5 (17–86)	54 (18–91)	0.040*	26 (17–67)	63 (34–91)	<0.001*
Flow ratio (Qp:Qs)	1.75 (0.85–15.1)	1.9 (0.41–13.77)	0.649	3.07 (1.53–15.1)	1.09 (0.41–2.24)	<0.001*
Anti-PH Therapy^a^			0.181			<0.001*
None	7 (19.4%)	10 (21.3%)		13 (31%)	0 (0%)	
PDE5-inhibitor	24 (66.7%)	23 (48.9%)		29 (69%)	19 (46.3%)	
PDE5-inhibitor + prostacyclin analog	5 (13.9%)	14 (29.8%)		0 (0%)	22 (53.7%)	
ECG Parameters						
P wave morphology in V1^a^			0.512			0.176
Normal	12 (33.3%)	18 (38.3%)		19 (45.3%)	11 (36.8%)	
Pulmonal	9 (25%)	7 (14.9%)		8 (19%)	8 (19.5%)	
Mitral	15 (41.7%)	22 (46.8%)		15 (35.7%)	22 (53.7%)	
P_II_	1.63 (1.62–2.93)	1.73 (0.08–3.11)	0.385	1.54 (1.62–2.90)	1.76 (0.08–3.11)	0.303
QRS_V1_	0.09 (0.04–0.13)	0.10 (0.06–0.19)	0.19	0.09 (0.04–0.16)	0.10 (0.06–0.19)	0.128
R_V1_	6.58 (0–24.5)	8.94 (0–32.19)	0.030*	4.95 (0–15.91)	12.47 (0–32.19)	<0.001*
R/S_V1_			0.219			0.031*
<1	5 (13.9%)	3 (6.4%)		7 (16.7%)	1 (2.4%)	
≥1	31 (86.1%)	44 (93.6%)		35 (83.3%)	40 (97.6%)	
S_V5_	7.10 (1.54–25.36)	11.37 (1.43–35.56)	0.07	5.61 (1.43–29.26)	15.37 (1.7–35.56)	<0.001*
S_V6_	5.46 (1.70–24.0)	10.19 (1.0–28.0)	0.132	4.23 (1.24–22.56)	12.02 (1.0–28)	<0.001*
RVSLI	13.76 (2.09–41.53)	25.72 (4.27–55.87)	0.051	11.78 (2.09–38.53)	29.17 (4.77–55.87)	<0.001*
PR_int_	0.18 (0.12–0.30)	0.19 (0.13–1.19)	0.276	0.19 (0.12–0.30)	0.19 (0.12–1.19)	0.645
PR_dep_			0.415			0.299
Yes	1 (2.8%)	3 (6.4%)		1 (2.4%)	3 (7.3%)	
No	35 (97.2%)	44 (93.6%)		41 (97.6%)	38 (92.7%)	
QRS axis	105.5 (39–167)	105 (15–143)	0.818	105 (15–167)	108 (56–143)	0.071
RV strain			0.573			<0.001*
Yes	20 (55.5%)	29 (61.7%)		13 (31%)	36 (87.8%)	
No	16 (44.5%)	18 (38.3%)		29 (69%)	5 (12.2%)	

^a^Comparison test on categorical data using Chi-squared or Fisher exact. ^b^Comparison test on continuous data using independent t test. ^c^Comparison test on continuous data using Mann–Whitney. *Statistically significant if p < 0.05. RVIDd: Right Ventricular Internal Diameter at end-Diastole, TAPSE: Tricuspid Annular Plane Systolic Excursion, LVEF: Left Ventricular Ejection Fraction, TR: Tricuspid Regurgitation, PVRI: Pulmonary Vascular Resistance Index, RVSP: Right Ventricular Systolic Pressure, PCWP: Pulmonary Capillary Wedge Pressure, SVR: Systemic Vascular Resistance, mPAP: Mean Pulmonary Arterial Pressure, PDE5-i: Phosphodiesterase type 5 Inhibitor, RVSLI: Right Ventricular Sokolow–Lyon Index, RAP: Right Atrial Pressure, PVR: Pulmonary Vascular Resistance

Median RAP was 8 (3–24) mmHg, and median PVR was 4.54 (0.08–45.86) WU. Patients in group PVR ≥ 5 WU showed worse TAPSE, so was higher PH probability, mPAP, PVRI, and RVSP (p < 0.05). All patients in this group received anti-PH therapy. In contrast, there was no significant difference of PH occurrence among all patients based on their RAP value.

### ECG parameters

**Fig. 1:**
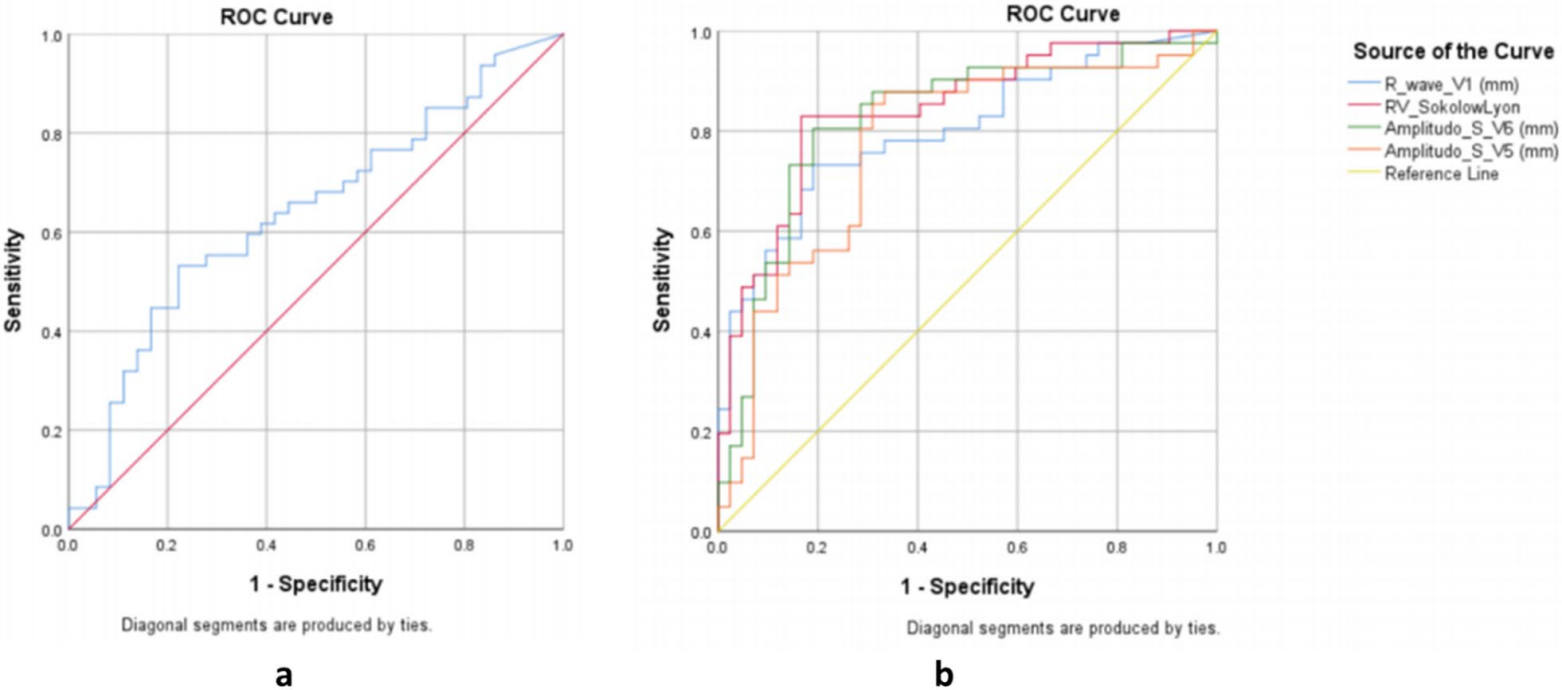
ROC curve of ECG parameters on RAP (**a**) and PVR (**b**)

From all ECG parameters, we compared based on RAP, R_V1_ was the only parameter found to be significantly different (p = 0.03). Furthermore, we obtained ROC curve to determine diagnostic value of R_V1_ on RAP as shown in Fig. 1a. The ROC of R_V1_ ≥ 7.5 showed that it had sensitivity 61.7% and specificity 61.1% in predicting RAP value ≥ 8 mmHg.

We performed similar analysis of ECG parameters based on PVR and RAP, respectively. Firstly, we performed comparison test, and then, we obtained ROC curve on those ECG parameters found to be significantly different from comparison test. Later, we performed bivariate analysis based on cut-off value obtained from ROC. As presented in Table 1, several ECG parameters were significantly different based on their PVR, namely R/S_V1_, R_V1_, S_V5_, S_V6_, RVSLI and RV strain. Figure 1b showed ROC curve of those continuous parameters on PVR. AUC of ECG parameters when they were divided by PVR value was all better than AUC of R_V1_ parameter based on RAP (Table 2).

**Table 2:** ROC curve analysis of continuous ECG variable based on univariate analysis

ECG parameters	AUC	95% CI		Cut-off	Sensitivity	Specificity
		Lower	Upper			
R_V1_^a^	0.639	0.519	0.760	7.5	61.7%	61.1%
R_V1_^b^	0.801	0.706	0.897	8.3	73.2%	81.0%
S_V5_^b^	0.773	0.668	0.878	7.4	80.5%	71.4%
S_V6_^b^	0.823	0.728	0.918	8.8	80.5%	81.0%
RVSLI^b^	0.841	0.755	0.927	16.1	82.9%	83.3%

^a^ROC analysis of ECG parameter on RAP, ^b^ROC analysis of ECG parameter on PVR, RVSLI: Right Ventricular Sokolow–Lyon Index, RAP: Right Atrial Pressure, PVR: Pulmonary Vascular Resistance

Bivariate analysis was carried out with Chi-square test, using the cut-off value obtained previously (Tables 3 and 4). Since several ECG parameters showed p value < 0.2 on PVR in bivariate analysis, these parameters were entered into multivariate analysis. Among all these ECG parameters analyzed, RVSLI (OR 15.66; 95% CI 4.46–55.02, p < 0.001) and RV strain (OR 9.23, 95% CI 2.43–35.14, p = 0.001) were found to be the best ECG parameter in predicting PVR ≥ 5 WU.

**Table 3:** Bivariate analysis of RV1 on RAP

ECG parameters	RAP < 8 mmHg		RAP ≥ 8 mmHg		p value	OR	95% CI	
	n	%	n	%			Lower	Upper
**R**_**V1**_					0.039*	2.53	1.04	6.17
< 7.5 mm	22	61.1	18	38.3				
≥ 7.5 mm	14	38.9	29	61.7				

^*^Bivariate analysis using Chi-squared, statistically significant if p < 0.05. RAP: Right Atrial Pressure

**Table 4:** Bivariate and multivariate analysis of ECG parameters on PVR

ECG parameters	Bivariate analysis^a^										Multivariate analysis^b^				
	PVR < 5 WU			PVR ≥ 5 WU			p value	OR	95% CI		B	p value	OR	95% CI	
	n		%	n		%			Lower	Upper				Lower	Upper
**R/S**_**V1**_							0.031*	8.00	0.94	68.26					
< 1	7	16.7		1	2.4										
≥ 1	35	83.8		40	97.6										
**R**_**V1**_							< 0.001*	11.59	4.12	32.62					
< 8.3	34		81.0	11		26.8									
≥ 8.3	8		19.0	30		73.2									
**S**_**V5**_							< 0.001*	10.31	3.71	28.66					
< 7.4	30		71.4	8		19.5									
≥ 7.4	12		28.6	33		80.5									
**S**_**V6**_							< 0.001*	17.53	5.89	52.18					
< 8.8	34		81.0	8		19.5									
≥ 8.8	8		19.0	33		80.5									
**RVSLI**							< 0.001*	24.29	7.70	76.63	2.751	< 0.001	15.66	4.46	55.02
< 16.1	35		83.8	7		17.1									
≥ 16.1	7		16.7	34		82.9									
**Strain RV**							< 0.001*	16.06	5.13	50.29	2.223	0.001	9.23	2.43	35.14
No	29		69.0	5		12.2									
Yes	13		31.0	36		87.8									

^a^Bivariate analysis using Chi-squared

^b^Logistic regression test in backward method

*significant in p < 0.05

RVSLI: Right Ventricular Sokolow–Lyon Index, PVR: Pulmonary Vascular Resistance

## Discussion

In this retrospective study, we found several ECG parameters were correlated with RAP and PVR. The RAP 8 mmHg and PVR 5 WU were taken as the cut-off in dividing study population since these two values were used as hemodynamic parameter thresholds in REVEAL score and ESC/ERS risk stratification system on PAH [5, 11]. COHARD-PH is a large registry of adult CHD population based in Yogyakarta, involving thousands of patients with various adult CHD. This study population had similar clinical characteristic with COHARD-PH, showing that our study may represent the common characteristic of adult CHD patients in Indonesia (female, young adult, developed PH) [12].

All patients in this study had normal range TAPSE values indicating proper RV contractility function; however, comparison test showed significantly difference TAPSE between study groups, both by RAP and PVR. Patients in group of RAP ≥ 8 mmHg and PVR ≥ 5 WU had lower TAPSE, indicating RV ejection fraction of patients in those groups began to decrease. In conditions where PVR increase chronically as in PH, RV dysfunction will occur when RV fails to adapt toward pressure overload. The adaptation of RV, by increasing muscle contractility and wall thickness, aims to maintain stroke volume without increasing RV filling pressure. However, RV contractility will be no longer be maintained, resulting in RV dilatation to produce equivalent stroke volume so that normal cardiac output is fulfilled [13].

In previous studies, examining ECG parameters on PAH population, ECG showed correlation with echocardiographic and functional parameters, yet those results were conflicting, probably due to various PAH population involved [6, 14–16]. (Our study involved only PAH population related to ASD). In the current study, R_V1_ was the only ECG parameters to be found significantly different based on RAP and PVR. However, the AUC obtained on RAP was only 0.639. This showed R_V1_ was considered poor in discriminating RAP groups. In contrast, the AUC obtained on PVR showed greater value overall. The R_V1_ ≥ 8.3 may predict PVR ≥ 5 WU with good sensitivity (73.2%) and specificity (81%). This result supported previous study by Sato et al., where they found reduced R_V1_ ≥ 1 mm after anti-PH therapy correlated with significant improvement of mPAP, PVR, RAP, and CI in PH patients. Nevertheless, only 22% of their study participants were CHD related [14].

We found no other ECG parameters significantly different based on RAP. This might be due to the RAP cut-off we used in the current study was lower compared to previous studies. Previous study by Mirtajaddini et al. in precapillary PH population showed that P pulmonal in V1 and PR_dep_ ≥ 1 mm correlated with RAP, and those 2 parameters showed satisfying sensitivity and specificity in predicting RAP value > 14 mmHg. Their study involved 77% patients PH group I, though it was not mentioned how many were suffering from CHD [10].

In general, PH resulting from CHD has better prognosis than IPAH or other types of PAH. This is due to the presence of intracardiac shunt, preventing syncope and cardiovascular collapse anytime PVR extremely increasing [17]. Normal RAP may occur in CHD accompanied by Eisenmenger syndrome, indicating RAP could be preserved to normal with the presence of intracardiac shunt, even though this will result in desaturation and cyanosis [18]. This is probably the reason why RAP and RVSP increase in PH resulted from CHD is not as severe as other types of precapillary PH. As in the current study, of all 83 patients enrolled, median RAP was 8 (3–24) mmHg and only 7 (8.4%) patients had RAP > 14 mmHg.

Recent study by Tokgoz et al. (22% of study population had CHD) found that QRS axis > 100° and R/S_V1_ > 0.9 predicted PVR > 2 WU with sensitivity 36.7%, 84% and specificity 93.4%, 40%, respectively [6]. Similarly, our study also found R/S_V1_ > 1 might predict PVR ≥ 5 WU with great sensitivity. Ninety percent of our patients had RAD, which is common in ASD. However, we found no significant difference of QRS axis based on both PVR and RAP. This could be due to the QRS axis in our study was analyzed as continuous data, not as categorical data like in previous studies, since we aimed to determine the cut-off value of QRS axis.

Prolonged QRS duration manifested as complete right bundle branch block (RBBB) was common in PH; it was proven to be correlated with worse prognosis and higher mortality risk [21]. Patients with PVR ≥ 5 WU and RAP ≥ 8 mmHg had longer QRS duration, yet this was not statistically significant. Only 18% of our study population had QRS duration > 120 ms. This result was similar to study by Sun et al. where they compared QRS duration based on mPAP, RAP, PCWP and PVR on patients with IPAH [7].

Prolonged PR interval manifested as 1st degree AV block was common in primum ASD, its occurrence in secundum ASD is likely to increase as patients get older and it reflects overload in right atrium. As far as we know, there has not been any study investigating PR_int_ correlation with hemodynamic parameter in PH or ASD previously, but one study by Tonelli et al. did find more prolonged PR_int_ from first-time diagnosed until approaching time of death on PAH patient [22]. However, this study found no significant difference of PR_int_ based on PVR and RAP, probably because our study population were young adults mostly.

Based on AHA recommendation in interpreting ECG (2009), S wave amplitude > 10 mm in V5, S wave amplitude and > 3 mm in V6, or RVSLI > 1.05 mV are some of criteria we can use in diagnosing RVH by ECG [19]. Our study found these parameters showed satisfying diagnostic value in predicting PVR ≥ 5 WU. This finding supported prior study by Igata et al., where they found RVSLI was significantly correlated with PVR, mPAP and RV mass index in precapillary PH [9]. Interestingly, their cut-off value was similar to our finding (16.1 vs 16.4 mm).

Extended T wave inversion reflecting significant pathological changing in cardiac geometry especially RV/LV volume ratio. Prior study by Miura et al. showed T wave inversion extended until V4 had sensitivity 69.2% and specificity 75.7% in predicting mPAP ≥ 25 mmHg in PAH and CTEPH [8]. Another study by Waligora et al. found extended T wave inversion until V5 correlated with severe RV dilatation and predicted worse survival rate in patients with PAH [23]. Unfortunately, we did not analyze how extensive the RV strain pattern in our study population. Yet in our study, this parameter along with RVSLI was found to be the best ECG parameters in predicting PVR ≥ 5 WU among adults with uncorrected secundum ASD.

This study has some limitations. First, this is a single-center study involving small size of study population. The lack of our study population might be caused by our catheterization laboratory restriction for elective procedure, including RHC, during COVID-19 pandemic. The second, we did not randomize our patients. However, the main strength of our study was its study population. As it was mentioned before that in developed country most CHD including ASD were corrected during childhood, moreover many previous studies on population with precapillary PH, including large registries of PH, involving only small portion of CHD patients.

## Conclusion

Our study demonstrated that RVSLI ≥ 16.1 mm and RV strain pattern had reliable diagnostic value, with great sensitivity and specificity in predicting PVR ≥ 5 WU. This finding supported ECG measurement as simple, noninvasive, and inexpensive screening modality in adults with uncorrected secundum ASD, especially in developing country or rural areas where health care facility and resources are limited.

## Supplementary Information

Supplementary material

## Data Availability

Data of the current study are available for further analysis upon reasonable request of corresponding author.

## Abbreviations

CHD: Congenital Heart Disease
ESC/ERS: European Society of Cardiology/European Respiratory Society
mPAP: Mean Pulmonary Arterial Pressure
PAH: Pulmonary Arterial Hypertension
PCWP: Pulmonary Capillary Wedge Pressure
PVR: Pulmonary Vascular Resistance
RAP: Right Atrial Pressure
REVEAL: Registry to Evaluate Early and Long-Term PAH Disease Management
RLHC: Right and Left-sided Heart Catheterization
RVSLI: Right Ventricular Sokolow–Lyon Index
