# Supplementary figures and images for: Electrocardiographic markers predict hemodynamic parameters in adults with uncorrected secundum atrial septal defect
Source: Egypt Heart J. 2025 Jan 10;77:8. doi: 10.1186/s43044-024-00596-x (PMC11723876; doi:10.1186/s43044-024-00596-x)

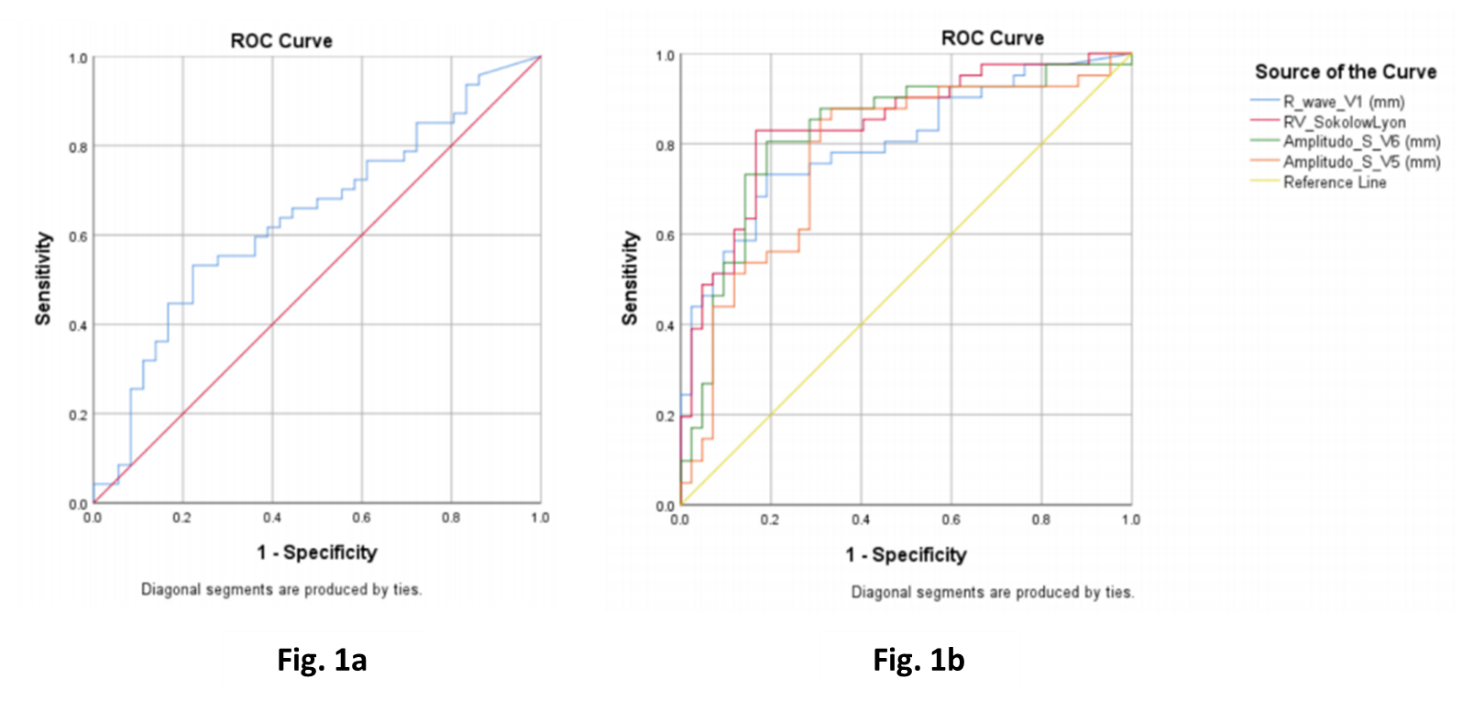


**Figure 1.** ROC curve of ECG parameters on RAP (1a) and PVR (1b)

Supplement: Supplementary file 1 — Supplementary material [file 43044_2024_596_MOESM1_ESM.docx]
